# Confusion Effects of Facial Expression Recognition in Patients With Major Depressive Disorder and Healthy Controls

**DOI:** 10.3389/fpsyg.2021.703888

**Published:** 2021-10-12

**Authors:** Fan Mo, Jingjin Gu, Ke Zhao, Xiaolan Fu

**Affiliations:** ^1^State Key Laboratory of Brain and Cognitive Science, Institute of Psychology, Chinese Academy of Sciences, Beijing, China; ^2^Department of Psychology, University of Chinese Academy of Sciences, Beijing, China

**Keywords:** facial expression recognition, major depressive disorder, confusion effect, negative emotions, discrimination sensitivity

## Abstract

Facial expression recognition plays a crucial role in understanding the emotion of people, as well as in social interaction. Patients with major depressive disorder (MDD) have been repeatedly reported to be impaired in recognizing facial expressions. This study aimed to investigate the confusion effects between two facial expressions that presented different emotions and to compare the difference of confusion effect for each emotion pair between patients with MDD and healthy controls. Participants were asked to judge the emotion category of each facial expression in a two-alternative forced choice paradigm. Six basic emotions (i.e., happiness, fear, sadness, anger, surprise, and disgust) were examined in pairs, resulting in 15 emotion combinations. Results showed that patients with MDD were impaired in the recognition of all basic facial expressions except for the happy expression. Moreover, patients with MDD were more inclined to confuse a negative emotion (i.e., anger and disgust) with another emotion as compared to healthy controls. These findings highlight the importance that patients with MDD show a deficit of sensitivity in distinguishing specific two facial expressions.

## Introduction

As important social cues from others, facial expressions are crucial for human interaction (Adolphs, 1999; Frank and Stennett, 2001). An accurate understanding of these non-verbal cues affects the efficiency of social interaction and underlies the satisfaction of interpersonal communication. Individuals with major depressive disorder (MDD), a mental disorder characterized by abnormal emotion processing, have been repeatedly reported to have difficulty in recognizing facial expressions (Bourke et al., 2010; Dalili et al., 2015 for reviews). The deficit in facial expression recognition is considered to be a critical factor for poor communication and alterations of adaptive behaviors in depressive individuals.

A notable cognitive theory of depression was proposed by Beck (1976) who theorized that depression was caused by negative cognitive schemata, such as themes of loss, separation, failure, worthlessness, and rejection. Numerous studies have demonstrated that depressed individuals tended to focus on the negative stimulus, which was congruent with their negative schemata (e.g., Mogg et al., 2006). Therefore, it might be the negative processing bias of depressed individuals that lead to the generation and persistence of depressive symptoms (Ramel et al., 2007). Reviewing 31 studies, Weightman et al. (2014) found that patients with MDD had difficulties in social interaction, which were partly caused by a reduced ability to correctly process emotional stimuli and interpret mental states. Gotlib and Joormann (2010) concluded that the key characteristics of depression include interpreting information negatively, having difficulties in disengaging from negative stimuli, and having deficits in cognitive control when depressed individuals processed negative information.

Some researchers explored the processing of facial expressions in depressed individuals, mainly focusing on the accuracy. However, the results were mixed, with some studies supporting the general deficit of patients with MDD (e.g., Asthana et al., 1998) and others in favor of an emotion-specific deficit (e.g., Bourke et al., 2010). For instance, Asthana et al. (1998) adopted simple pattern identification, facial pattern identification, and facial component discrimination tasks to examine whether perceptual impairment in depressed individuals is general, or specific, to all or certain emotional categories, using happy, sad, fear, and angry emotions. Using the mixed factorial design with repeated measures, they found that patients with MDD performed worse than general medical patients or healthy controls in the emotional discrimination task, but the emotional recognition impairment of depressed individuals was not specific to certain types of emotions. Similarly, Persad and Polivy (1993) used facial affective and questionnaire booklet to measure all the emotional recognition responses of female participants and found that both depressed psychiatric and depressed college students made more overall errors, which were not emotion-specific, in recognizing facial expressions (i.e., fear, anger, disgust or contempt, sadness, indifference, surprise, and happiness), than healthy controls.

On the contrary, some studies were in favor of a deficit in emotion-specific recognition for patients with depression. For example, depressed patients had an impaired ability to distinguish emotional expressions, especially happiness and sadness, compared with healthy controls (e.g., Mikhailova et al., 1996). Mikhailova et al. (1996) aimed to explore the recognition of emotional facial expressions in patients with both MDD and schizotypal personality disorder (STP). In their study, sad, happy, and neutral faces were followed by a masking stimulus. These faces were displayed for 80 ms randomly in the left or right hemifield. They found that patients with MDD showed a serious impairment in recognizing sadness and happiness in comparison with healthy controls. Similarly, Surguladze et al. (2004) also found the lower recognition accuracy in identifying happy expressions in patients with MDD, compared with healthy controls. They examined the recognition accuracy and response bias toward positive and negative facial expressions in patients with unipolar depression and healthy controls (Surguladze et al., 2004). Participants with MDD were asked to label each facial expression as happy, sad, or neutral. Calculating with the discrimination accuracy and response bias scores (i.e., a higher response bias score indicates a stronger tendency to misidentify neutral faces as emotional), the results showed a lower accuracy in identifying happy expressions and a conservative response bias to happy facial expressions relative to the sad facial expressions in patients with depression. The possible reason for the emotion-specific bias may be the tendency that depressed individuals judged social interactions or situations more negative or less positive.

Unlike the deficit of sad facial expression recognition in depressed individuals, some researchers found that depressed participants were more accurate in identifying sad facial expressions than healthy volunteers (Gollan et al., 2010). For instance, Gollan et al. (2010) asked participants with MDD and healthy control with no psychiatric illness to judge the emotional types of facial expressions by pressing one of six colored keyboard keys. Happy, surprised, angry, sad, fearful, and disgusted emotions were morphed to produce an expression, which displayed ranging from 10 (90% neutral) to 80% of the emotion. Facial expressions were presented for 500 ms and in 10% increments to generate a range of intensities. Participants with depression outperformed healthy controls in recognizing sad facial expressions. Moreover, the relationship between the facial recognition accuracy and severity of depressive symptoms indicates that as depressive symptoms became more serious, the recognition accuracy for sad facial expressions increased while the recognition accuracy for surprised facial expressions decreased, in line with previous research showing that depressed individuals had better performance in recognizing sad facial expressions due to the congruency of the emotional information with depressive disorder (Rusting, 1998). Furthermore, excluding the studies using schematic faces, neuroimaging studies, and drug treatment and synthesizing findings across a total of 22 studies on the facial emotion recognition in depressed individuals and healthy controls, a meta-analysis study showed emotional recognition impairment existed in all basic emotions except of sadness (Dalili et al., 2015).

When required to label the emotion category of a facial expression, participants misattributed and confused the emotion with another one, and this phenomenon in recognizing facial expression has been investigated in children (Gagnon et al., 2010; Young, 2014) and healthy adults (Roy-Charland et al., 2014). A possible explanation for the confusion between emotions might be attributed to shared action units and visual similarities of facial expressions (Camras, 1980; Wiggers, 1982). Ekman and Friesen (1978) proposed a Facial Action Coding System (FACS) that defined the muscle activation of facial expressions, which was used to code the single facial muscles. For example, a happy facial expression is characterized by the raise of angulus oris (AU6) and cheek (AU12). Roy-Charland et al. (2014) found that participants had difficulty in discriminating fearful and surprised facial expressions, which they attributed to the similar visual configurations of fearful and surprised facial expressions. Besides confusion between surprise and fear, Young (2014) also examined the confusion of distinguishing facial expressions between disgust and anger in children. They found that children easily confused these expressions not only due to their visual similarities of these facial expressions but also because children did not allocate their attention to facial regions equally. Facial expressions involved different muscle movements; thus, some emotion pairs that shared more action units and more similar facial muscle movements are easier to be confused with each other than other emotion pairs. Although the confusion effect of facial expressions was pervasive in recognizing facial expressions, the confusion effect of facial expression recognition in patients with MDD has not been systematically explored for all six basic emotions.

The six-alternative forced choice (6AFC) task is a widely used paradigm in the field of facial expression recognition. Participants were asked to identify the emotional expression by pressing one of six keys that listed each of six emotions (i.e., happiness, surprise, disgust, sadness, fear, and anger) (Schaefer et al., 2010). However, the 6AFC task is not suitable to explore confusion effects in distinguishing between two emotions, such as sadness-anger or happiness-surprise, which makes it impossible to measure the discrimination sensitivity to a specific emotion pair. Unlike the 6AFC task, a 2AFC task could provide us direct evidence about the confusion effects of specific emotion pairs and could enable us to compare the confusion effects of specific emotion pairs between different subject samples.

Utilizing the pairwise comparison method (i.e., a 2AFC task), the goals of this study were twofold as follows: (1) to examine the overall accuracy and reaction time of facial emotion recognition for six basic emotions (i.e., happiness, anger, disgust, sadness, surprise, and fear) in patients with MDD and healthy controls and (2) to compare the confusion effects for each emotion pairs between patients with MDD and healthy controls.

## Materials and Methods

### Participants

The participants with MDD were recruited from the Zhumadian Psychiatric Hospital in China. The inclusion criteria for the depressed patients were as follows: (1) at the age of 16–33 years; (2) native Chinese; (3) right-handed; (4) primarily diagnosed as unipolar MDD, according to the *Diagnostic and Statistical Manual of Mental Disorders* (Fourth Edition, DSM-IV); (5) scores of Hamilton Depression Scale (HAMD) 17≥17; and (6) not taking psychiatric medication for 2 months or not taking the psychiatric medication regularly.

We excluded patients satisfying any of the following criteria: (1) comorbid with other mental disorders; (2) comorbid with other serious physical diseases; (3) having a history of the cerebral organic disease; (4) having a history of cerebration injury; (5) receiving electrical shock treatment; (6) pregnant or lactating women; (7) having a history of alcohol and substance abuse; (8) claustrophobia; and (9) intellectual disability.

Thirty patients with MDD (17 females; age: *M* = 24.23 years, *SD* = 5.82) and 30 healthy participants (15 females; age: *M* = 21.90 years, *SD* = 2.14) were recruited in this study. Two patients with MDD were excluded because one was admitted to the hospital with alcohol dependence and the other turned manic after a week. Finally, 28 patients with MDD and 30 healthy participants were included for further analysis. The demographic and clinical information of patients with MDD and healthy controls after removing the two patients with MDD are summarized in Table 1. The detailed clinical scores and demographics of patients with MDD are shown in Supplementary Material (Table S1).

**Table 1:** Demographic and clinical information of major depressive disorder (MDD) patients and healthy controls.

	**Patients with MDD**	**Healthy controls**	**Independent-sample *t*-test**
*N*	28 individuals	30 individuals	
Age (*M ± SD*)	24.29 ± 5.67	21.9 ± 2.14	*t*_(56)_ = −2.15, *p* = 0.036
Gender	12 males; 16 females	15 males; 15 females	—
Illness duration (years)	2.01 ± 1.94	—	—
Education	11.61 ± 2.85	15.57 ± 1.85	*t*_(56)_ = 6.32, *p <* 0.001
HAMD score (*M* ±*SD*)	23.11 ± 3.89	2.97 ± 2.53	*t*_(56)_ = −23.54, *p* < 0.001
HAMA score (*M* ±*SD*)	18.64 ± 7.72	—	—

*HAMD, Hamilton Depression Scale; HAMA, Hamilton Anxiety Scale*.

### Stimuli

The stimuli included 60 images of six basic emotional facial expressions from the Ekman database, posed by 10 human models of whom four were males and six were females (Ekman and Friesen, 1976). The study protocol, based on Ekman and Friesen's Brief Affect Recognition Test (Ekman and Friesen, 1974), was modified in our previous study (Zhao et al., 2017). Six basic emotional facial expressions (i.e., fear, surprise, anger, disgust, sadness, and happiness) were examined in pairs, resulting in 15 emotion pairs in total.

### Procedure

**Figure 1:**
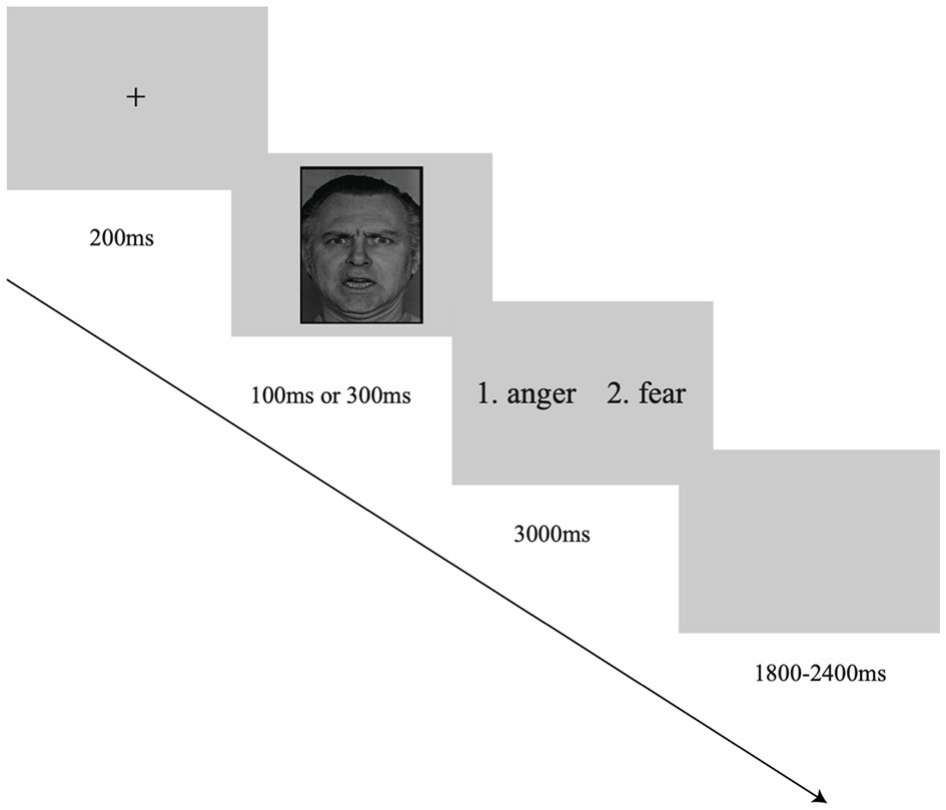
The illustration of a single trial in the facial expression recognition task. The facial expression image was obtained from the Ekman database (Ekman and Friesen, 1976).

In each trial, a fixation was initially presented for 200 ms, followed by a facial expression image on the screen for 100 or 300 ms. Participants were required to identify the emotion from the presented image and to respond by performing a 2AFC task. Specifically, they needed to choose “1” or “2” (e.g., 1 = anger and 2 = fear) by pressing the corresponding key as quickly and accurately as possible. The interstimulus interval (ISI) randomly ranged from 1,800 ms to 2,400 ms (Figure 1). The whole study consisted of 15 blocks, and each of which had 20 trials. The trials of two different facial expressions were equally presented in each block. Moreover, the sequence of blocks was random, and the presentation of emotional stimuli was also in random order. There were four practice trials before the formal experiment.

### Data Analysis

First, we calculated the average recognition accuracy and reaction time for each facial expression. The repeated measures ANOVA (rmANOVA) was conducted on the reaction time and recognition accuracy, respectively, with the expression category (i.e., anger, happiness, sadness, fear, surprise, and disgust) and group (patients with MDD vs. healthy controls) as the independent variables and the reaction time or the recognition accuracy as the dependent variable. Then, we calculated the discrimination sensitivity (d′) to evaluate the ability to discriminate from every emotional facial expression pair, with the hit and false alarm specific to each expression within each pair according to the signal detection theory. For example, for a block consists of angry and happy facial expressions, the Hit refers to choosing “anger,” the Miss refers to choosing “happiness” when presented an angry face, the False Alarm refers to choosing “angry,” and the Correct Rejection refers to choosing “happiness” when presented a happy face. The d′ score is calculated as the *Z*-score for a hit (*Z*_*H*_) minus the *Z*-score for a false alarm (*Z*_*FA*_), i.e., ${\;d}^{\prime }=Z_{H}-Z_{FA}$.

This calculation method of d′ was also used in previous studies (e.g., Galvin et al., 2003; Sweeny et al., 2013; Zhao et al., 2017; Koeritzer et al., 2018). For example, in the field of memory research, Koeritzer et al. (2018) presented sentences to participants and required them to report whether they had heard the sentence before. In their study, the d′ was adopted to assess the extent to which participants could discriminate between old and new items. In the field of facial expression recognition, the d′ has been adopted as the discrimination sensitivity index. Zhao et al. (2017) explored the neural response to facial expressions of fear and surprise in an emotional recognition task. They calculated the sensitivity of discrimination between the two categories of facial expressions.

Consistent with the previous studies as stated earlier, we also adopted d′ as a sensitivity index to assess the extent to which participants can discriminate between two emotional facial expressions (e.g., happiness and sadness). The discrimination sensitivity index was calculated for 15 emotion pairs. The independent *t*-tests were then conducted to compare the discrimination sensitivities between patients with MDD and healthy controls for each emotion pair, with d′ as the dependent variable. We also calculated the correlation coefficient of the discrimination sensitivity between patients with MDD and healthy controls, aiming to explore whether patients with MDD and healthy controls had similar confusion patterns in 15 emotion pairs or not.

Besides the discrimination sensitivity index (d′), we also calculated the confusion matrix based on the number of accurately recognizing emotional facial expressions. If all the participants recognized the facial expressions correctly, the total numbers of recognizing each specific facial expression such as happiness were 1,400 (28 participants × 10 trials × 5 pair groups) for all patients with MDD and 1,500 (30 participants × 10 trials × 5 pair groups) for all healthy controls, and the total numbers of recognizing different facial expressions in each emotion pair were 280 (28 participants × 10 trials) for all patients with MDD and 300 (30 participants × 10 trials) for all healthy controls. According to the Hit Rate, Miss Rate, False Alarm rate, and Correct Rejection Rate of each emotion pair, we calculated the Recall Ratio, Precision Ratio, and F_1_ score as follows: $\text{Recall}=\frac{\text{hit rate}}{\text{hit rate + miss rate}}$, $\text{Precision}=\frac{\text{hit rate}}{\text{hit rate + false alarm rate}}$, and F1=2×Precision×RecallPrecision+Recall. In our study, for example, for the happiness-sadness emotion pair, the Precision Ratio is the proportion of the sad recognition accuracy rate in the actual sad emotional recognition rate plus happy emotional recognition rate. The Recall Ratio is the proportion of sad emotional recognition accuracy rate in the rate of actual sad emotional expressions. A larger F_1_ score, which ranges from 0 to 1, indicates a greater ability to discriminate between two emotions.

## Results

### Discrimination Sensitivity

**Figure 2:**
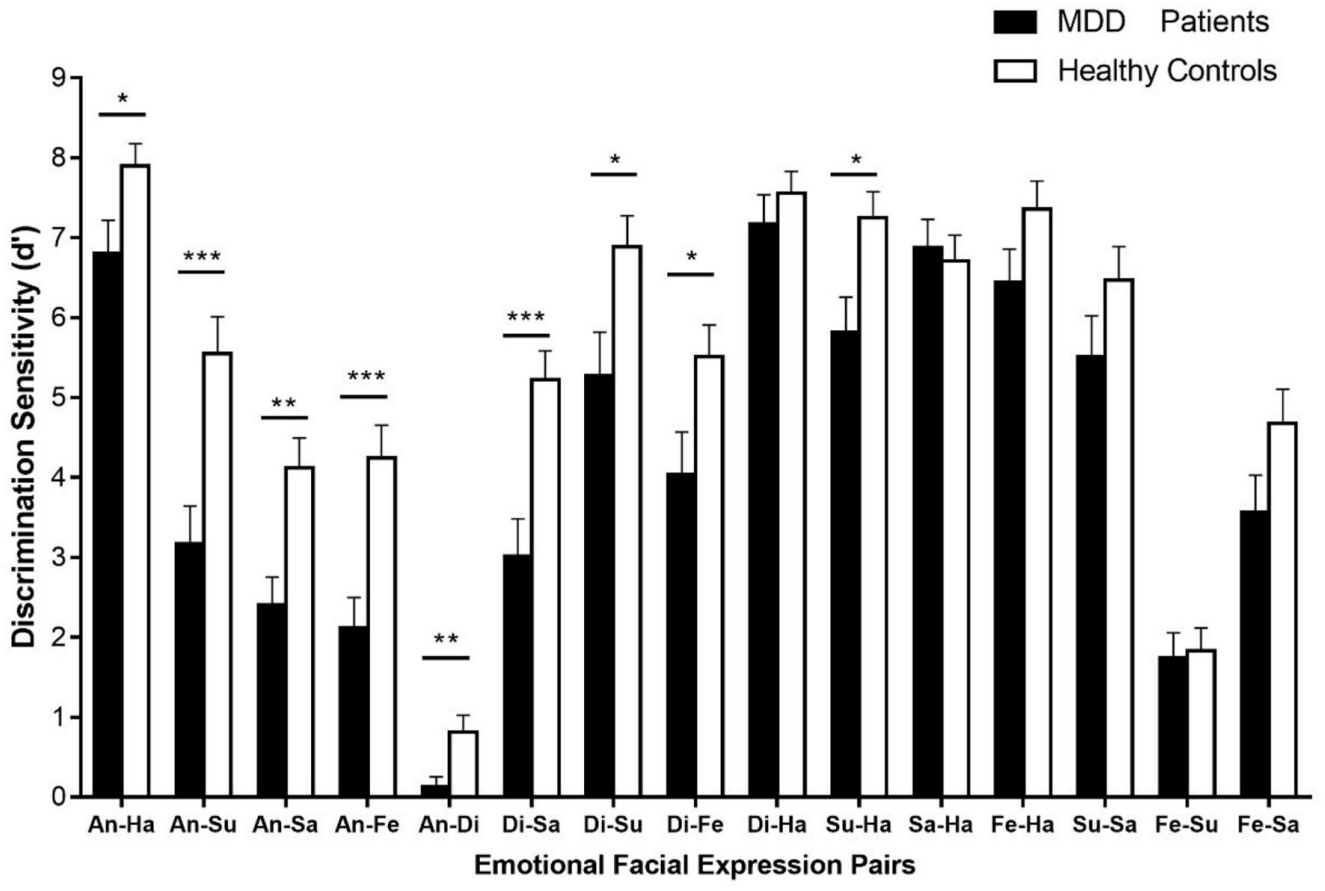
The discrimination sensitivities (d′) for 15 emotional facial expression pairs. Error bars represent SEMs. Di, disgust; An, anger; Fe, fear; Su, surprise; Sa, sadness; Ha, happiness. *p < 0.05; **p < 0.01; ***p < 0.001.

The descriptive statistics of the discrimination sensitivity are shown in Supplementary Material (Table S2). The independent sample *t*-tests were conducted to compare the discrimination sensitivity (d′) of 15 emotional expression pairs between patients with MDD and healthy controls (Figure 2). It showed that the discrimination sensitivity of patients with MDD was significantly smaller than those of healthy controls in various emotional facial expression pairs, including disgust-anger [*t*_(56)_ = −2.71, *p* < 0.01], sadness-anger [*t*_(56)_ = −3.36, *p* < 0.01], fear-anger [*t*_(56)_ = −3.77, *p* < 0.001], sadness-disgust [*t*_(56)_ = −3.81, *p* < 0.001], fear-disgust [*t*_(56)_ = −2.23, *p* < 0.05], surprise-anger [*t*_(56)_ = −3.61, *p* < 0.01], surprise-disgust [*t*_(56)_ = −2.45, *p* < 0.05], surprise-happiness [*t*_(56)_ = −2.65, *p* < 0.05], and happiness-anger [*t*_(56)_ = −2.22, *p* < 0.05]. However, the discrimination sensitivities between two groups showed no difference in pairs of sadness-happiness [*t*_(56)_ = 0.38, *p* > 0.05], happiness-disgust [*t*_(56)_ = −0.87, *p* > 0.05], surprise-sadness [*t*_(56)_ = −1.45, *p* > 0.05], fear-sadness [*t*_(56)_ = −1.76, *p* > 0.05], fear-happiness [*t*_(56)_ = −1.70, *p* > 0.05], and fear-surprise [*t*_(56)_ = −0.21, *p* > 0.05].

**Figure 3:**
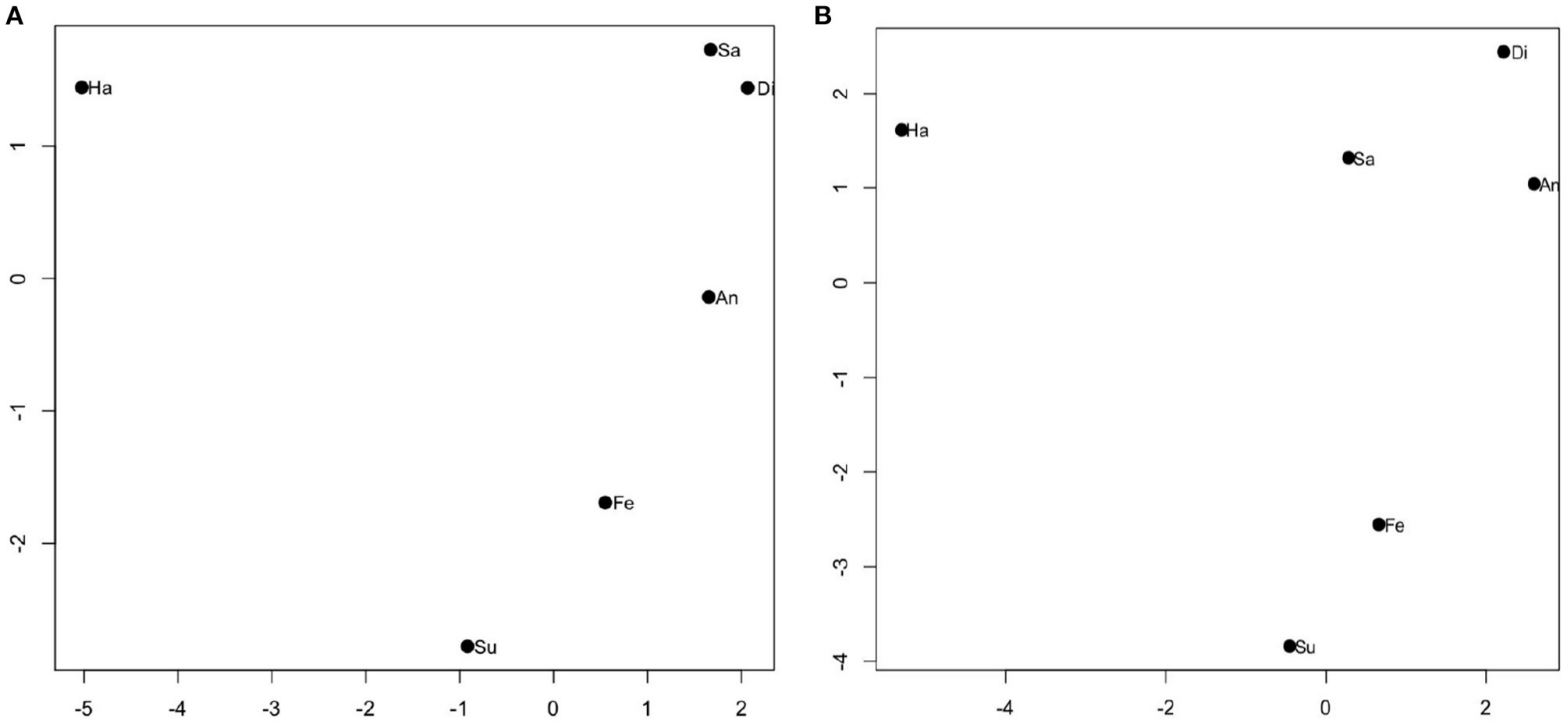
The multidimensional scaling (MDS) solution, based on the discrimination sensitivity (d′) values. The horizontal and vertical coordinates have no special meaning, while the period between the two emotions reflected the discrimination sensitivity value. Di, disgust; An, anger; Fe, fear; Su, surprise; Sa, sadness; Ha, happiness. **(A)** The discrimination sensitivity values for major depressive disorder (MDD) patients and **(B)** the discrimination sensitivity values for healthy controls.

**Figure 4:**
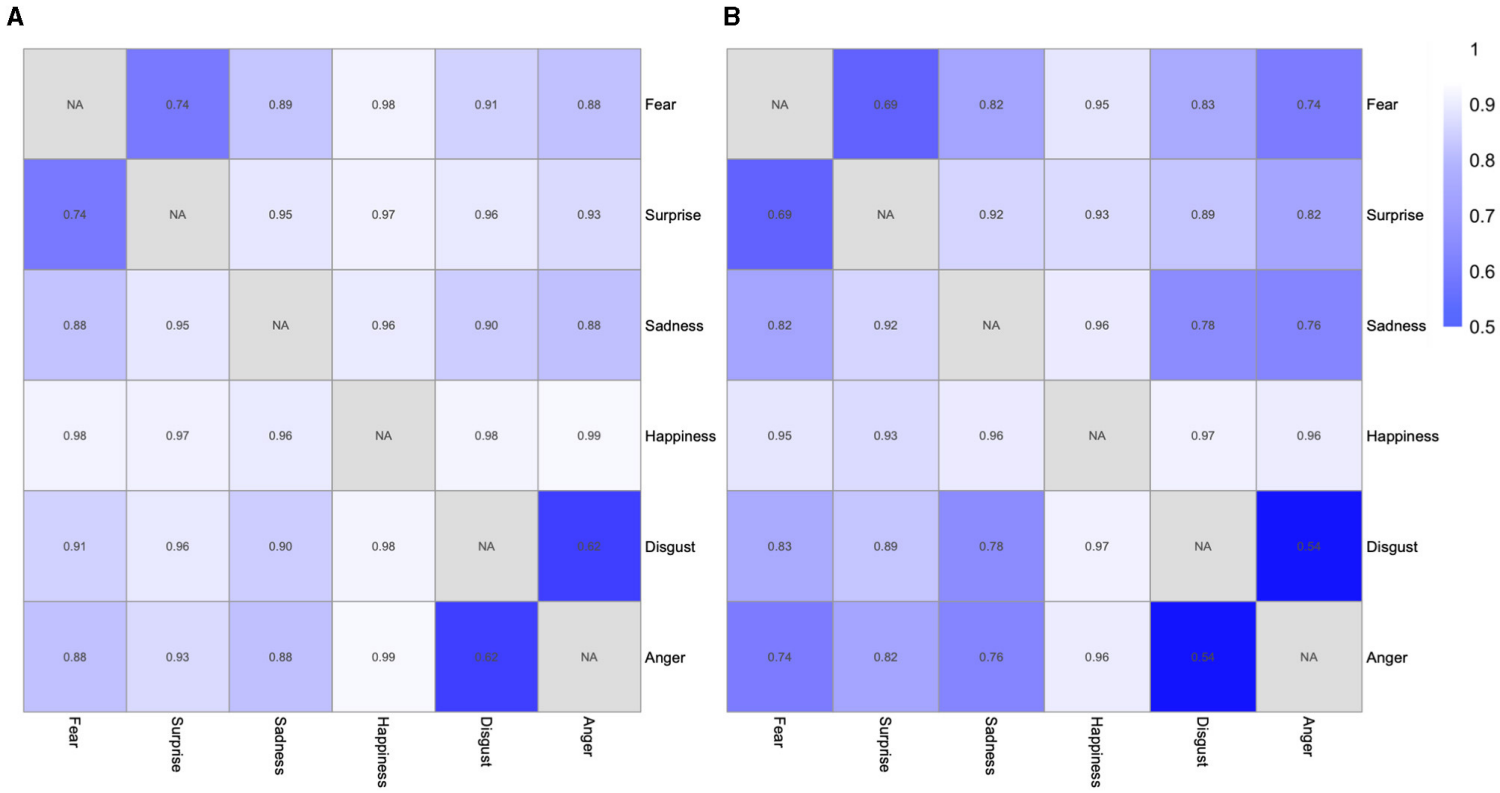
The heat map of the F_1_ scores of recognizing facial expressions. **(A)** The heat map of the F_1_ scores of recognizing facial expressions of 15 emotional pairs for healthy controls. **(B)** The heat map of the F_1_ scores of recognizing facial expressions of 15 emotional pairs for patients with MDD.

We further used the multidimensional scaling (MDS) to show the magnitude of discrimination sensitivity, which is reflected in the different periods between two emotions (Figure 3). As shown in Figure 3, the positive emotion (i.e., happiness) was relatively far away from the other expressions, indicating that happiness was easier to be distinguished from other emotions. As shown in Tables 2, 3, for example, the “1,122” in the second column and second row in Table 2 means the total number of responses for “Fear” when the fearful facial expression was presented for 28 participants with MDD. The “88” in the third column and second row in Table 2 means the total number of responses for “Surprise” when the fearful facial expression was presented for 28 patients with MDD. The results of the confusion matrix of recognizing the facial expression of emotions in patients with MDD and healthy controls showed that fear was more likely to be confused with surprise and disgust was more likely to be confused with anger (Tables 2, 3). Consistent with this confusion matrix, it was easier for both patients with MDD and healthy controls to distinguish between happiness and other emotional expressions; however, it was more difficult to distinguish between fear and surprise and between anger and disgust. As shown in the heat map of the F_1_ scores (Figure 4), the lighter the color is, the easier it is to distinguish between two expressions (e.g., happiness-anger). We also found that anger-disgust and fear-surprise were the two easily confused emotional pairs.

**Table 2:** The confusion matrix of recognizing facial expressions for patients with MDD.

	**Fear**	**Surprise**	**Sadness**	**Happiness**	**Disgust**	**Anger**
Fear	1,122	88	55	18	52	65
Surprise	74	1,222	16	18	35	35
Sadness	35	31	1,220	5	65	44
Happiness	8	20	18	1,339	8	7
Disgust	36	25	56	7	1,154	122
Anger	81	69	104	14	142	990

**Table 3:** The confusion matrix of recognizing facial expressions for healthy controls.

	**Fear**	**Surprise**	**Sadness**	**Happiness**	**Disgust**	**Anger**
Fear	1,326	71	32	9	31	31
Surprise	90	1,372	11	6	10	11
Sadness	36	18	1,375	12	35	24
Happiness	5	10	12	1,462	8	3
Disgust	18	13	22	4	1,339	104
Anger	43	35	54	4	128	1,236

### Correlation Analysis

**Figure 5:**
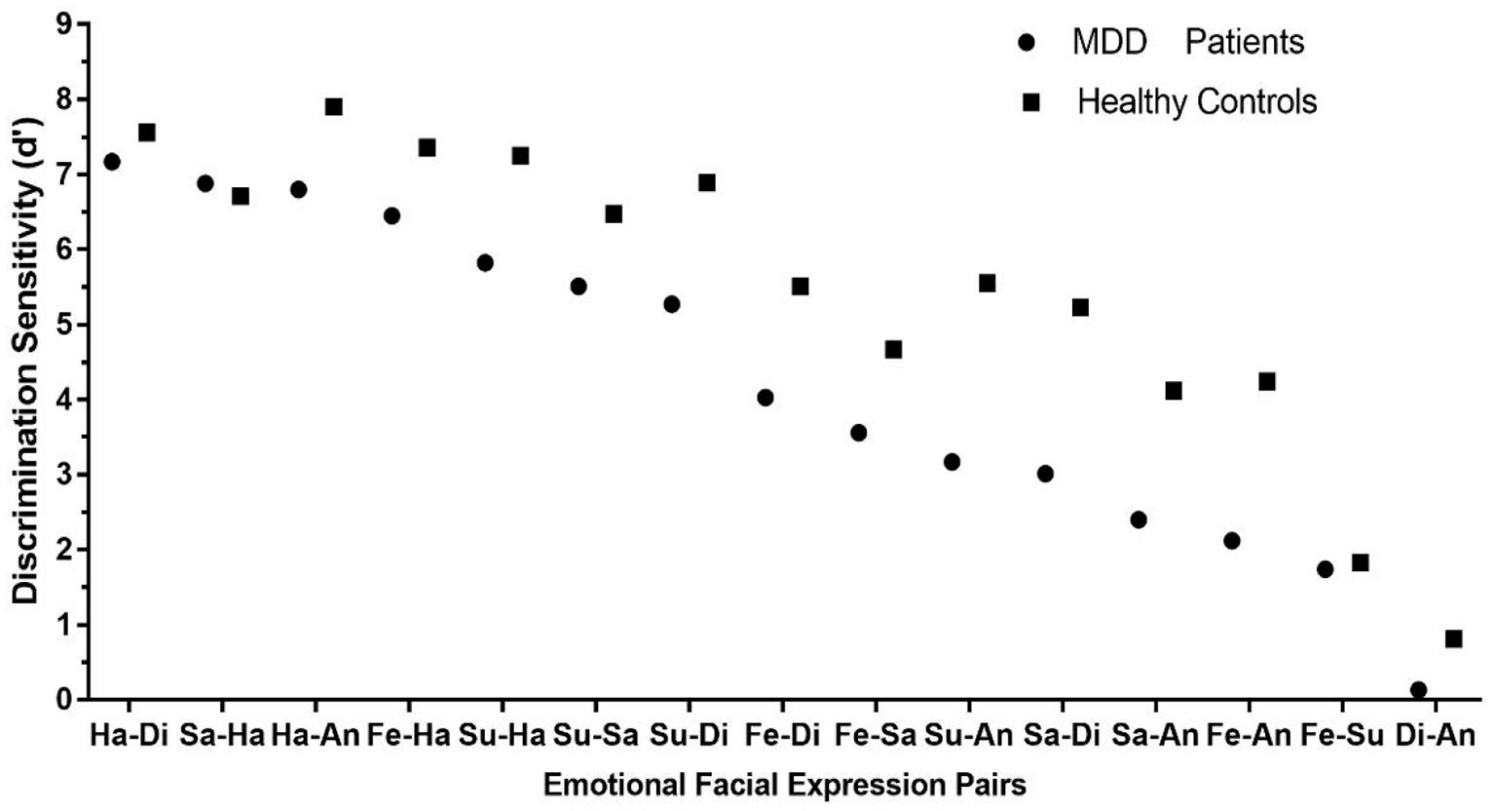
The scatter diagram of discrimination sensitivities (d′) in patients with MDD and healthy controls.

The Spearman's correlation revealed a strong correlation of the discrimination sensitivity between patients with MDD and healthy controls, *r* = 0.929, *p* < 0.01, indicating that patients with MDD and healthy controls showed a similar pattern in discriminating different facial expression pairs. Figure 5 shows the scatter diagram of discrimination sensitivities (d′) of 15 emotional facial expression pairs in patients with MDD and healthy controls.

### Recognition Accuracy

**Figure 6:**
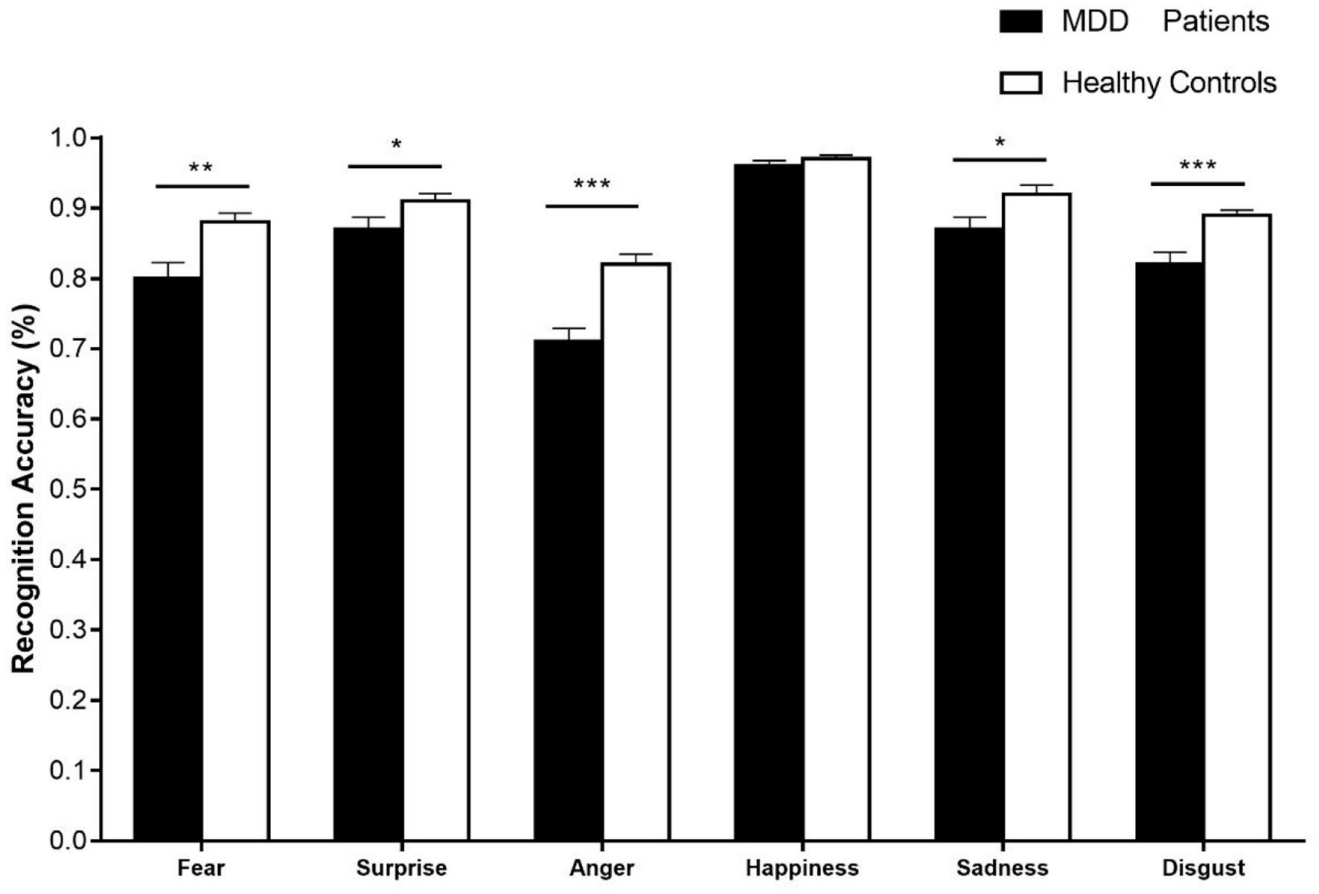
The recognition accuracies of six basic emotional expressions in patients with MDD and healthy controls. Error bars represent SEMs. *p < 0.05; **p < 0.01; ***p < 0.001.

The descriptive statistics of recognition accuracy is shown in Supplementary Material (Table S3). A 2 × 6 rmANOVA was conducted on the recognition accuracy with emotion category (i.e., fear, anger, disgust, surprise, sadness, and happiness) as a within-subject factor and group (i.e., patients with MDD and healthy controls) as a between-subject factor. The main effect of emotion category was significant, [*F*_(4.19,234.73)_ = 66.52, *p* < 0.001, $\eta_{p}^{2}$ = 0.54]. The main effect of group was also significant, [*F*_(1, 56)_ = 19.95, *p* < 0.001, $\eta_{p}^{2}$ = 0.26]. In addition, the interaction between two factors was significant, [*F*_(4.19,234.73)_ = 4.58, *p* < 0.01, $\eta_{p}^{2}$ = 0.08]. The simple effect analyses showed that the recognition accuracies between patients with MDD and healthy controls significantly differed in surprise (*p* < 0.05), fear (*p* < 0.01), anger (*p* < 0.001), disgust (*p* < 0.001), and sadness (*p* < 0.05). The recognition accuracy of happy facial expression showed no difference between patients with MDD and healthy controls (*p* > 0.05). These results are shown in Figure 6.

### Reaction Time

**Figure 7:**
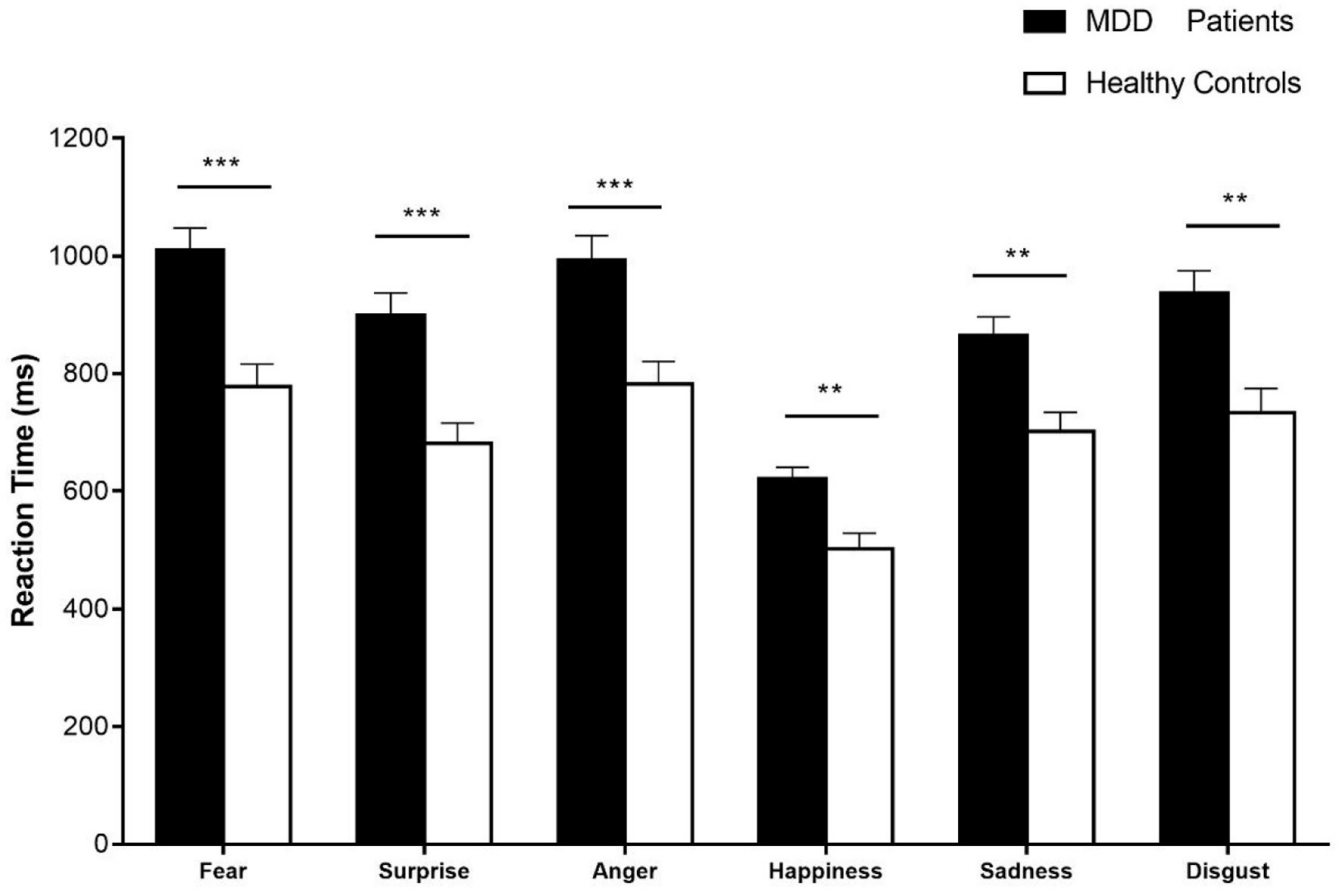
The reaction time of six basic emotional expressions in patients with MDD and healthy controls. Error bars represent SEMs. *p < 0.05; **p < 0.01; ***p < 0.001.

The descriptive statistics of reaction time are shown in Supplementary Material (Table S4). A 2 × 6 rmANOVA was conducted on the reaction time with emotion category (i.e., fear, anger, disgust, surprise, sadness, and happiness) as a within-subject factor and the group (i.e., patients with MDD and healthy controls) as a between-subject factor. There was a significant main effect for emotion category, [*F*_(4.11, 230.02)_ = 93.21, *p* < 0.001, $\eta_{p}^{2}$ = 0.63], and a significant main effect for group, [*F*_(1, 56)_ = 18.58, *p* < 0.001, $\eta_{p}^{2}$ = 0.25]. In addition, the interaction between two factors was significant, [*F*_(4.11, 230.02)_ = 2.76, *p* < 0.05, $\eta_{p}^{2}$ = 0.05]. The simple effect analyses showed that the reaction time of patients with MDD and healthy controls significantly differed in surprise (*p* < 0.001), fear (*p* < 0.001), anger (*p* < 0.001), disgust (*p* < 0.01), happiness (*p* < 0.01), and sadness (*p* < 0.01). The results are shown in Figure 7.

## Discussion

In this study, we used a 2AFC task to investigate the confusion effects of facial expression recognition in patients with MDD and healthy controls. The confusion effect refers to the phenomenon that one emotion is confused with another emotion in facial expression recognition. It is measured by the d′, indicating the degree of difficulty to distinguish two emotional facial expressions. We found that the confusion effects of facial expression recognition for emotion pairs that included negative emotions (i.e., anger and disgust) were stronger in patients with MDD than in healthy controls. More specifically, compared with healthy controls, patients with MDD were more inclined to confuse anger with disgust, fear, sadness, surprise, and happiness. Also, these patients had greater difficulty in discriminating between disgust and other negative expressions such as sadness, surprise, and fear. Furthermore, the correlation analysis of discrimination sensitivity (d′) for 15 emotion pairs showed a similar pattern of confusion phenomenon between patients with MDD and healthy controls. Particularly, disgust was confused with anger and fear was confused with surprise among 15 emotion pairs both in patients with MDD and in healthy controls. In addition, patients with MDD showed lower recognition accuracy for sadness, fear, surprise, disgust, and anger. No difference of the recognition accuracy was found in recognizing happiness between patients with MDD and healthy controls. Patients with MDD had a longer reaction time in recognizing all facial expressions than healthy controls, suggesting that they need more time than healthy controls to judge the types of emotions.

The main aim of this study was to compare the difference of confusion effects in recognizing emotional facial expressions between patients with MDD and healthy controls. When participants performed a 2AFC task, they were required to identify the emotional category of a human face displaying on the screen and to show their answers by pressing one of the two keys. This paradigm allowed us to assess the ability to discriminate two emotional facial expressions, using the analysis of discrimination sensitivity index (d′). Our results revealed a profound confusion effect of recognizing facial expressions that were specific to the emotion pairs such as anger with other emotions for patients with MDD, compared with healthy controls. More specifically, patients with MDD were mostly inclined to confuse anger with the other five basic emotional facial expressions (i.e., happiness, sadness, surprise, disgust, and fear). A reason for this result could be due to the attributes of angry facial expression. The angry facial expression of others was a negative and threatening stimulus, signaling that “something is wrong” or “danger is approaching” (Burklund et al., 2007). According to the social risk (SR) hypothesis, the depressive phenomenon can be conceived as defensive psychobiological responses to increased risk, for example, depressed individuals would be hypersensitive to signals of social threats from others, signal to others for reducing SRs, and inhibit risk-seeking behaviors (i.e., the inhibition of confident and acquisitive behaviors) (Allen and Badcock, 2003). Therefore, when patients with MDD were asked to identify the facial expression involving angry emotion, they were more inclined to confuse the risky emotional stimuli (e.g., angry facial expressions) with another emotional stimulus. The confusion phenomenon could also be explained by the hypothesis that depression is related to an inhibition of the emotion of anger (Riley et al., 1989). Seidel et al. (2010) measured the automatic behavioral tendencies in response to angry, fearful, sad, happy, and neutral facial expressions for depressed patients and healthy controls, using an implicit joystick task. It revealed that only depressed patients showed pronounced avoidance tendencies in response to angry faces, reflecting a stronger response of the aversive motivational system, which could support our results that showed a strong confusion effect of recognizing angry facial expressions when anger was paired with another emotion for patients with MDD. Therefore, our results could be explained as resulting in part from the avoidance tendency when depressed individuals processed angry faces; similarly, it was also found that depressed participants did not exhibit attentional biases to angry faces. However, individuals with generalized anxiety disorder were more likely to firstly observe threatening faces, compared with healthy controls and individuals with depressive disorder (Mogg et al., 2000; Gotlib et al., 2004). As discussed earlier, depressed patients, who perceive angry faces as more threatening than healthy controls (Gollan et al., 2008), tend to avoid angry stimuli, which results in a strong confusion effect of facial expression recognition when anger was paired with another emotion for patients with MDD.

Another possible explanation for the stronger confusion with angry pairs for patients with MDD could be the reduced ability to recognize angry facial expression. For example, in previous research which employed a morphed stimuli paradigm, participants were asked to rate the intensity of displayed images with happiness, anger, sadness, disgust, and fear. It was found that depressed patients were less accurate in decoding the emotion of anger at 70% of intensity than anorexic patients and healthy controls (Mendlewicz et al., 2005). Therefore, the lower accuracy in anger recognition for patients with MDD may result in a larger confusion effect of recognizing facial expressions in patients with MDD when anger was paired with another emotion, compared with healthy controls.

According to the discrimination sensitivity index, our results also showed that pairs of anger-disgust, disgust-sadness, disgust-surprise, and disgust-fear were more difficult to be distinguished in patients with MDD when compared with healthy controls, which mainly focused on the confusion of disgust with another emotion. An expression of disgust, similar to that of anger, was also a negative and threatening stimulus (Rozin and Fallon, 1987). Therefore, the possible reason of the results was that depression is related to the inhibition of the emotion of disgust, as the interpretation of the results of the confusion effect of angry discussed earlier. Another possible explanation could be the smaller accuracy of disgusted facial expression recognition in patients with MDD. For example, the previous study examined facial expression processing in patients with severe depression and healthy control groups, using a modified version of the facial expression recognition task. The depression group displayed a specific deficit in the facial expression recognition of disgust, which may be related to impaired functioning of frontostriatal structures, especially the basal ganglia (Douglas and Porter, 2010). In addition, processing the threatening emotional stimuli consumed attention resources (Pessoa, 2005). Therefore, the consumption of attention resources for patients with MDD might contribute to the confusion of distinguishing disgust from other emotions (i.e., sadness, surprise, fear, and anger).

In addition, the pattern of discriminating different pairs of facial expressions in patients with MDD and healthy controls was similar, which is supported by the evidence that the results showed a significantly positive correlation in discrimination sensitivity (d′) of 15 emotion pairs between patients with MDD and healthy controls. Furthermore, as shown in the results of the confusion matrix and the heat map of the F_1_ scores, disgust-anger was the most difficult pair to be distinguished among 15 pairs of emotional facial expressions both in patients with MDD and in healthy controls. Fear was easily confused with surprise in patients with MDD and healthy controls. These results indicate that it is easier to confuse the two expression pairs for patients with MDD and healthy controls, in line with previous studies (e.g., Young, 2014). The theoretical reasoning for the results that fear was confused with surprise and anger was confused with disgust was the hypothesis of the perceptual-attentional limitation. These results of confusions may arise from a difficulty in perceiving the difference between two facial expressions, or a lack of attention to distinguish two facial expressions (Roy-Charland et al., 2014). The difficulty in distinguishing may be caused by similar visual configurations of two facial expressions. In particular, both fear and surprise involve the activation of the inner brow raiser, outer brow raiser, upper lip raiser, and jaw-dropping. Anger and disgust also share some action units, involving the activation of the lip raiser, the lip part, and the chin raiser. Contrary to difficulty in distinguishing between fear and surprise and between anger and disgust, it is easier to discriminate between happiness and other emotional expressions because happiness is quite different from other emotional facial expressions in action units. Besides the similar visual configurations, the confusion of surprise and fear might be explained by the stimuli novelty, because surprise- and fear-eliciting events are typically appraised as unexpected, which was not the case for other emotions (Vrticka et al., 2014).

We found in patients with MDD an overall impairment in recognizing all expressions except for happiness. Furthermore, our findings showed that the reaction time of recognizing six basic expressions in patients with MDD was significantly longer than healthy controls, which echoes with evidence that supporting the deficit of the patients with MDD in processing emotional expressions leads to impaired interpersonal functioning (Surguladze et al., 2004). The longer reaction time could be explained by the fact that depressed individuals performed retarded on cognitive tasks (Williams et al., 1988). The impaired ability to think or concentrate in patients with MDD underlies the general cognitive impairment and mental operation reduction in these patients (Asthana et al., 1998). Thus, the longer reaction time in recognizing facial expressions for patients with MDD is likely to reflect a more general perceptual-motor deficit rather than the specific effect on processing facial expression (Persad and Polivy, 1993). Moreover, our experiment task, in which participants were asked to press the corresponding key when the briefly presented emotional stimuli disappeared, required participants to allocate their attention resources. Actually, depressed individuals have broad difficulty with concentration and memory (Burt et al., 1995). Therefore, individuals with depression make more errors in emotional recognition and show a longer reaction time in identifying emotional stimuli.

It should also be noted that this study did not find impairment in the recognition of happy expression, for both patients with MDD and healthy controls. This might be attributed to the differences in facial configurations between happy facial expression and other expressions. A happy facial expression includes the raise of angulus oris (AU6) and cheek (AU12) (Ekman and Friesen, 1978), which is quite different from other negative facial expressions. Therefore, a happy face includes different muscular movements from other emotional facial expressions, leading it to be easily discriminated from other basic facial expressions.

Our study has some limitations. First, we only employed a behavioral index for the difference of confusion effect between patients with MDD and healthy controls. Future studies could examine the neural mechanism underlying the difference in the confusion effect. Second, some demographic variables were not strictly controlled, such as the education level. Third, the number of adolescent patients with MDD was only 3, so we could not compare adolescent and adult participants with MDD in subgroups. Further studies need to compare the differences in recognizing facial expressions in adolescents and adults. Finally, this study focused on emotional facial expressions but omitted neutral expressions. Future studies should incorporate emotionally neutral expression to further explore the difference in the confusion effect between patients with MDD and healthy controls.

## Conclusion

By adopting the 2AFC paradigm, current findings underscore the importance of understanding the deficit in recognizing facial expressions for patients with MDD and highlight the role that the strong confusion effect in recognizing facial expressions between negative emotions (i.e., anger and disgust) and other specific emotions for patients with MDD might be an indicator for the detection of depression.

## Data Availability Statement

The raw data supporting the conclusions of this article will be made available by the authors on reasonable request.

## Ethics Statement

The studies involving human participants were reviewed and approved by the Ethics Committee of Beijing Huilongguan Hospital and the Ethics Committee of Zhumadian Psychiatric Hospital (Ethic Approval No: 2016-72). Written informed consent to participate in this study was provided by the participants, and where necessary, the participants' legal guardian/next of kin.

## Author Contributions

FM: conceptualization, methodology, software, data analysis, writing—original draft preparation, reviewing, and editing. JG: writing—reviewing and editing. KZ: conceptualization, methodology, writing—reviewing and editing, and supervision. XF: supervision and writing—reviewing and editing. All authors contributed to the article and approved the submitted version.

## Funding

This work was supported by National Natural Science Foundation of China (32071055 and 62061136001) and National Social Science Foundation of China (19ZDA363).

## Conflict of Interest

The authors declare that the research was conducted in the absence of any commercial or financial relationships that could be construed as a potential conflict of interest.

## Publisher's Note

All claims expressed in this article are solely those of the authors and do not necessarily represent those of their affiliated organizations, or those of the publisher, the editors and the reviewers. Any product that may be evaluated in this article, or claim that may be made by its manufacturer, is not guaranteed or endorsed by the publisher.
