# Supplementary material for: Confusion Effects of Facial Expression Recognition in Patients With Major Depressive Disorder and Healthy Controls
Source: Front Psychol. 2021 Oct 12;12:703888. doi: 10.3389/fpsyg.2021.703888 (PMC8546352; doi:10.3389/fpsyg.2021.703888)
Supplement: Supplementary file 1 [file Data_Sheet_1.PDF]

Table S1. The clinical scores and demographics of the MDD patients

Patients	Gender (1-male; 2-female)	Age	HAMD scores	HAMA scores	Education (years)	Illness Duration (years)
2001	1	26	24	14	16	2
1003	1	29	22	15	9	3
2003	2	18	17	20	12	5
1004	1	20	22	15	9	2
2002	1	27	22	11	15	0.5
1006	2	18	25	9	12	2
1007	2	31	29	17	9	6
1009	1	26	25	16	9	6
1011	1	33	20	10	9	0.33
2006	2	17	18	10	12	0.17
1012	2	30	21	15	16	0.5
1014	2	20	18	14	16	0.75
1017	1	27	22	16	9	1
1018	2	19	25	16	13	4
1019	2	18	26	41	13	3
1020	1	24	18	11	9	0.17
1022	1	18	24	25	12	0.25
2008	2	33	25	30	12	0.5
1023	2	33	25	16	9	6
1024	2	18	18	11	12	1
1025	2	23	18	18	15	3
1028	1	25	22	21	16	2
1029	2	27	32	33	16	0.17
1030	2	30	31	21	7	1.33
1031	1	27	24	23	9	0.5
1032	1	30	24	30	9	0.25
1033	2	16	23	25	10	4
1034	2	17	27	19	10	0.75

Table S2. The mean discrimination sensitivity (d') of 15 emotional pairs in MDD patients and healthy controls

	MDD patients ($M \pm SD$)	Healthy Controls ($M \pm SD$)
An-Ha	6.80 ± 2.20	7.90 ± 1.52
An-Su	3.17 ± 2.48	5.55 ± 2.54
An-Sa	2.40 ± 1.86	4.12 ± 2.05

An-Fe	2.12 ± 2.00	4.24 ± 2.25
An-Di	0.13 ± 0.66	0.81 ± 1.15
Di-Sa	3.01 ± 2.49	5.23 ± 1.93
Di-Su	5.27 ± 2.90	6.89 ± 2.11
Di-Fe	4.03 ± 2.84	5.51 ± 2.17
Di-Ha	7.17 ± 1.95	7.56 ± 1.49
Su-Ha	5.82 ± 2.30	7.25 ± 1.79
Sa-Ha	6.88 ± 1.85	6.71 ± 1.77
Fe-Ha	6.45 ± 2.15	7.36 ± 1.93
Su-Sa	5.51 ± 2.71	6.47 ± 2.30
Fe-Su	1.74 ± 1.66	1.83 ± 1.55
Fe-Sa	3.56 ± 2.47	4.67 ± 2.37

Di=Disgust; An=Anger; Fe=Fear; Su=Surprise; Sa=Sadness; Ha=Happiness.

Table S3. The mean recognition accuracy of six emotions for MDD patients and healthy controls

	Happiness	Anger	Fear	Surprise	Sadness	Disgust
MDD patients (*M* ± *SD*)	0.96 ± 0.04	0.71 ± 0.10	0.80 ± 0.12	0.87 ± 0.09	0.87 ± 0.09	0.82 ± 0.09
healthy controls (*M* ± *SD*)	0.97 ± 0.03	0.82 ± 0.08	0.88 ± 0.07	0.91 ± 0.06	0.92 ± 0.07	0.89 ± 0.04

Table S4. The mean recognition reaction time of six emotions for MDD patients and healthy controls

	Happiness	Anger	Fear	Surprise	Sadness	Disgust
MDD patients (*M* ± *SD*) (ms)	621.56 ± 100.75	993.88 ± 214.89	1010.06 ± 196.23	899.76 ± 198.80	865.12 ± 165.84	733.75 ± 225.06
Healthy controls (*M* ± *SD*) (ms)	501.35 ± 150.48	781.86 ± 212.94	777.88 ± 209.02	680.64 ± 195.44	701.92 ± 177.75	936.41 ± 202.78
